# Autoethnographic Reflections on Mental Distress and Medication Management: Conceptualising Biomedical and Recovery Models of Mental Health

**DOI:** 10.1007/s10597-024-01230-5

**Published:** 2024-02-22

**Authors:** Joanna Fox

**Affiliations:** School of Allied Health and Social Care, Anglia Ruskin University, Cambridge, UK

**Keywords:** Expertise-by-experience, Autoethnography, Biomedical model, Recovery model, Shared decision-making

## Abstract

This article uses autoethnography to explore the author’s lived experiences of mental distress and how she has conceptualised and explained these symptoms to herself using both the biomedical and recovery models of care. Autoethnography is a process of personal reflection that enables connection between the personal and the political. Experiences of mental distress are recounted alongside the decision to reduce medication. This personal experience is then explored in the context of limited evidence base on the effectiveness of reducing medication and the situation in which prescribers often feel reluctant to recommend and support service users in these choices. Shared decision-making in medication management is introduced which is an approach which draws on the models of recovery and co-production challenging traditional biomedical approaches which locate the prescriber as expert. Moreover, the radical service user led model is highlighted, within which, the Hearing Voices Network and Open Dialogue offer alternative approaches which promote co-production and empowerment. The author connects the personal to the political and reflects on her dual identity as an expert-by-experience and social work academic. She details how she has drawn on biomedical explanations to describe her distress yet has been challenged by the recovery model throughout her journey of recovery. She concludes that her own position, in identifying herself as an academic and expert-by-experience is an important step in challenging notions of expertise and approaches to mental health care.

## Introduction

There are many approaches to mental health treatment and care, most often in the form of prescribed medication or a limited selection of specific evidence-based talking therapies such as Cognitive Behaviour Therapy (CBT). However, such treatment approaches are often deemed by people with lived experience to be unacceptable and inappropriate to their needs (Moncrieff, 2020; Watts et al., 2021); thus, failing to support their recovery. This can lead some service users to designate themselves as *survivors* of the mental health system, rather than as *consumers*. The biomedical model is often the predominant mental health framework in use by professionals. This framework leads to many implicit assumptions about the origin of mental ill health, the causes of symptoms, and consequently the best options for the care and treatment plans. Thus, within this model, for people experiencing psychosis, medication is still often the first line of treatment (Ceraso et al., 2020; Huhn et al., 2019).

Until the mid-twentieth century in western society, people experiencing mental health conditions were contained in asylums where professionals had the power to hold or release them. In the 1960s, people accessing services (Reaume, 2002) began to challenge the politics of mental health care. The development of normalisation, the principles of social role valorisation^1^ (Wolfensberger, 1972) and concomitant moves towards care in the community were associated with the shifting power balance in care and treatment. This slow but perceptible shift of power in the treatment of people experiencing mental ill-health (Reaume, 2002) was further driven by the conception of the social model of disability (Oliver, 1996) and the growing rejection of the medical model of mental health care Despite these movements, the past historical legacies of the asylums continue to haunt the experiences of many people today who access care and support. Moreover, some service users continue to experience serious side effects from taking medication for a number of years and professionals still have the power to compulsorily detain people in hospital and treat people against their will, under certain conditions set out in UK mental health legislation (Mental Health Act, 2007).

Building on the activism generated by the survivor movement, the recovery model (Deegan, 1996) emerged in the 1990s; it acknowledges the role of the person using services as the author of their own recovery. The personal model of recovery has been described in much literature, perhaps most notably in Leamy et al. (2011) seminal CHIME model of recovery, which covers five components of effective recovery-oriented services and interventions: Connectedness, Hope, Identity, Meaning and Empowerment. These components draw on a social model of mental health (Tew et al., 2012), emphasising the importance of factors such as employment, volunteering and friendship in living a meaningful life (Wood & Alsawy, 2018). Despite the development of the recovery concept within the user paradigm (Coleman, 1999; Deegan, 1996), increasingly over recent years it seems to have been subsumed into a professional clinical paradigm in which recovery outcomes are based on functional abilities and the absence of psychiatric symptoms; indeed, a version of this paradigm underpins much of mental health policy. Thus, despite activism of the user-led and survivor movement, treatment is most often being delivered within the auspices of the biomedical framework.

However, notwithstanding the limitations of the recovery frameworks, policy makers (DH, 2019) and practitioners (Ramon et al., 2021) aspire to promote service user agency and choice, with a focus on recovery, and to offer wider access to different forms of treatment. The NHS Mental Health Implementation Plan 2019/2020 (DH, 2019, p. 8) in England highlights the importance of co-production, stating that mental health plans should be developed and reviewed ensuring ‘Engagement and co-production with local communities, people with lived experience of mental ill health and mental health services, their families and carers, evidenced throughout the plan and included in continued governance structures’. This commitment places lived experience at the centre of the development, planning and implementation of mental health services in England. Thus, in mental health care and treatment, professionals increasingly claim to place precedence on service users’ rights to define their own experiences of recovery (Wood & Alsawy, 2018) and to agency in treatment planning (Ramon et al., 2021). Despite this, the mental health system is often experienced as deficit-led with a chronic shortage of resources limiting care provision to only either the most unwell, or to those who are deemed to be at risk of harm.

The process of reflection described in this article allows me to unpack assumptions about the biomedical and recovery models in my own journey of mental health recovery. The lived experience of psychosis and the trajectory of a mental health condition is distinctive to each person. Moreover, the symptoms of mental ill health, their severity and the outcomes people have, are hugely variable; thus, this narrative is based on my own lived encounters. Moreover, the way I have used medication, and experienced support from professionals, are also unique (Fox, 2021a). This article combines reflexive narratives shifting between perspectives of theory, story and practice, drawing upon autoethnography to frame this discussion. Autoethnographic writing is often controversial and often seen as methodologically of limited validity in the hierarchy of evidence (Murad et al., 2016) because it draws on lived experience; however, this stance is challenged by current mental health policy (DH, 2019) that privileges co-production and expertise-by-experience. Thus, autoethnographic writing has a place in enabling connections to be made between individual experience and academic knowledge (Fox, 2016).

I intentionally recorded reflections on my experiences of taking and reducing medication from December 2022—March 2023, because I wanted to share this lived experience perspective of taking medication. I wanted to reduce the side effects of my medication and explore if the strategies developed by other peers in taking medication might be helpful for me. Alongside this systematic recording of ideas and reflections, I continued to read about this topic developing and conceptualising theory as I alternatively wrote and read. This generated theoretical constructs from my reflections as I connected autobiographical material with the wider context of mental health care and support. Moreover, the art of autoethnographic writing (Adams et al., 2015) requires the researcher to pay careful attention to both the *epistemic* (claims to knowledge) and the *aesthetic* (practices of imaginative, creative, and artistic craft) characteristics of their texts as they seek to convey the meaning of their individual experiences and communicate their significance to the wider community of practice. Autoethnography is a method increasingly used in health and social care research (Amas & Fox, 2023) because it can facilitate the connection of “the autobiographical and personal to the cultural, social, and political” (Ellis, 2004). Hence, this article allows us to understand how diverse models of care can facilitate but also hinder a person making sense of their experiences of mental distress.

Thus, this article seeks to illuminate the often-hidden decision-making processes that service users make about accessing treatment. It aims to explore a subliminal space in which treatment regimens can be considered from lived experience perspective, a frame in which experiences are recorded relatively rarely. Finally, the article provides a reflection on the impact of the medical model on treatment and influence of the recovery model on care and support, leading to a consideration of the impact of interventions such as Open Dialogue and the Hearing Voices Network.

## Lived Experiences of Mental Distress

Throughout my journey of recovery, I have heard voices and had unusual experiences; they are often distressing and encountered as persecutory in nature (Woods et al, 2015). Such voices in my head stretched me like a rubber band, with the distress completely overwhelming my mind. This reflection captures that story:

*I heard voices inside my head that were real and authentic. I would lie in bed for hours ‘talking’ through my head to other students in other rooms in the halls of residence. I would hold in depth conversations with them. But then the conversations got darker, and the content more frightening, accusing me of things I hadn’t done or telling me to do things I needed to do that clashed with my moral code. Clashed with my faith and what I believed in. They were persistent, accusatory, de-valuing and highly critical, reducing me to a shell of my former self.*

The content of the voices became darker and more threatening as I was increasingly drawn into their world, losing touch with reality. They named things from the recesses of my mind. I had to identify them as existing outside of my control and separated them from me. I chose to identify them as a chemical imbalance. Thus, by using the medical model, I could denote the terminology of ‘hallucinations’, a label which allowed me to designate them as being part of an illness, rather than being part of me. However, the application of the biomedical model reinforced a sense of shame at the imposition of a new ‘service user identity’ (Fox, 2021b), rather than of being a student, an employee, or a relative. Moreover, both for me and for other people experiencing mental ill health, the loss of a sense of identity and more so of a sense of self, generated when accessing treatment within such a mindset, can be linked to the self-stigmatisation that many people with mental health issues experience (Corrigan & Rao, 2012). Thus, for me, reliance on the medical model was oxymoronic in that it both reinforced the pathology of my individual condition but simultaneously relieved self-stigmatisation as I felt less to blame for the development of my condition.

In contrast with this, Keogh et al. (2022) reports that individuals develop personal conceptualisations of voice-hearing, rejecting biomedical explanations and replacing them within a more holistic frame. As such, Hart (2021) advocates that is important to use the terminology of voice-hearing, rather than that of audio-visual hallucinations because it ‘dignifies’ this event for voice-hearers by adding credence to their experiences. In my case, I became committed to the medical model ‘excusing’ my own perceived guilt by blaming them on insufficient chemical messengers. Thus, I located existence as ‘without’ rather than ‘within’, dissociating part of the identity I had from encountering these experiences. I believed that anti-psychotics alone could address these symptoms; and eventually the voices were eradicated in their most vocal state by taking medication.

My next reflection illustrates the power of voices in drawing me into their reality, reflecting on the insidiousness of their nature.

*These voices had faces and I could assign them to people. It would surreptitiously compel me to listen as I was drawn into their worlds. But when I looked out of the door of my room, the people I had imagined talking weren’t there. I would succumb to hours of listening to these voices as I succumbed to their attraction. The voices would pull me in and secretly attract me. After a while they reduced. But I remained attracted to the surreptitious power of these voices.*

Moving on from the immediacy of my mental health crisis to the current day, as part of my long-term recovery strategy, I have taken medication for over 35 years to support my wellbeing and participated in CBT based therapies on several occasions. I have rarely questioned my decision to take medication or use evidence-based talking therapies because they have kept me relatively well, despite the side effects. Although it can be proffered that both treatment approaches offer a model in which mental ill-health is perceived to be a disorder that needs curing to make a person function more effectively in the accepted roles of society, indeed, some survivors experience traditional talking therapies, such as CBT and its derivatives, to be limited and focused on individualised deficiency. However, I experienced talking therapies to be effective (Fox, 2021c) because they were delivered by therapists using diverse eclectic approaches which could be fitted to my needs. However, both medication and talking therapies did fit my framing of the medical model in which my behaviour needed modifying, within my own deficit-focussed image of myself.

Recently I have re-read narratives of recovery told by service users from their perspective (Baker, 2009; Coleman, 2021); they have led me to think about my understanding of medication. I have looked again at the side effects of the medication: the higher levels of sedation, increase in weight, restless legs, propensity to develop diabetes; factors which many service users find to be intolerable. Increasingly I am compelled to reflect on my own decisions to take medication and access treatment within the medical model.

Medicinal sedation limits and boundaries my mind, however I have begun to reduce it; a moment of reflection in time reveals this experience:

*As I reduce medication, my head organisation seems to be crumbling and I no longer feel as organised. I feel distracted as if all things are coming at me. On reflection, with a dog walk, I can see that this is linked to a reduction in medication. Medication is something that dulls the senses and the stress as well. Now there are many things hitting me, I can’t deal with them one by one, it feels as if they are randomly coming at me. I feel as if my head is more open to the elements, that it’s not self-contained and organised with each arrangement logical and clear.*

In this process, I have once again explored the recovery model and mused about its application to my own story. I wonder, as I think, if recovery has lost its soul; and as I ponder about this, I should review my own conception of mental health and the treatment choices I have made.

## Discussion

In this next section I reflect on the different frames that can be used to understand mental health and can lead to treatment choices. I reflect on how I began to look outside the restrictive medical model of care to fully embrace a recovery model. I am a social work academic and an expert-by-experience (Fox, 2016); I have been accepted in that world as such. My professional expertise as a social work academic has given me an intellectual space to write extensively about how I have used mental health services (Fox, 2016, 2021a, 2021c) as a *consumer*.

As I reflect on my own status, and my commitment to the medical model, I fear that I have become estranged from my roots, leading to an over-conceptualisation of the recovery approach, and that my professionalism has taken precedence over my expertise-by-experience. I worry more widely that such representations have reduced this service user led radical construct (Coleman, 1999; Deegan, 1996) to a professional framework of service provision. I have viewed myself to be a consumer of mental health services, taking control of my medication regime with the support of mental health prescribers (Fox, 2021a). Medication is often effective in reducing distress, improving coping skills and ameliorating social and occupational functioning in people who have been diagnosed with schizophrenia (Ceraso et al., 2020; Huhn et al., 2019). However, Moncrieff (2020) draws on extensive evidence to demonstrate that psychiatric drugs do not ‘treat’ or ‘cure’ mental illness by acting on hypothesised chemical imbalances or other abnormalities in the brain, recognising that the positive effects of the drugs can come at great cost to people’s physical health and their ability to function in day-to-day life. The provision of medication is based on the construction of mental distress as a ‘psychotic disorder’, a label which can be both stigmatising and a source of ‘othering’ (Keogh et al., 2022). Recently, I have once again reduced my medication, a decision which many service users note is often challenged by professionals (Cooper et al., 2019). Hui et al. (2018) concluded that patients, who experienced a first episode of psychosis but responded fully to initial treatment, possessed a decreased risk of relapse and of better long-term clinical outcomes if they continued medication for the first three years after starting treatment compared to people who did not. They recommended that if patients opted to discontinue medication, they should receive adequate support and monitoring to reduce the potential of relapse.

Despite many professionals’ commitment to the importance of medication in maintaining mental wellbeing (Fleming et al., 2021), increasingly, methods of shared decision-making (SDM) in medication management (Fox, 2021a; Ramon et al., 2021) have been advocated to respect service users as experts in their own care. This is a process which breaks down the medical model of care, that views the professional as the expert, and reinforces the importance of co-production in mental health services (DH, 2019/2020). However, despite the emphasis on service user agency, Pappa et al. (2021), who undertook an online co-produced survey of SDM in medication management, found that although both patients and prescribers reported some good practice in collaborative prescribing, service users perceived clinicians to be less collaborative than they themselves reported. Moreover, Angell and Bolden (2015) noted how clinical prescribers used their professional power to persuade service users to be compliant with medication regimes. Furthermore, professionals often feel reluctant to reduce service users’ medication (Cooper et al., 2019) because of limited evidence in how to reduce it effectively often believing it to be safer to maintain medication usage, rather than risk any potential for relapse.

Such a stance emphasises the gap between the recovery model which advocates the self-management of service user’s own recovery, alongside the imposition of power and authority endowed in the clinician in the biomedical model of care. Likewise, given the reluctance of some clinicians to reduce service user’s medication (Cooper et al., 2019; Pappa et al., 2021), and the lack of power and respect they feel in controlling treatment choices, many service users choose to reclaim that power and reduce their medication by themselves without seeking the advice of professionals. Watts et al. (2021) reported that this lack of support led service users in their study to become ‘active agents in resisting the hegemonic, dominant narrative of a lifetime of taking medication’ (p. 1402). Additionally, Keogh et al. (2022) reported that ceasing / reducing medication was rarely a reactive decision based on lack of insight but was undertaken as a cost benefit analysis (Cappleman et al., 2015; Le Geyt et al., 2017), in which choices were made in terms of side effects and impact on the recovery. This indicates how mental health professionals (Keogh et al., 2022), who focus on concepts such as non-compliance and non-adherence to medication regimes often underestimate service users’ capacity to make choices.

Moreover, research into the reduction / cessation of medication treatment is new and innovative and leads to the biomedical model being challenged in whole systems of psychiatry. Oedegaard et al. (2020) have studied the introduction of medication-free services into a region in Norway. Increasing choices in the treatment options required the development of personal agency from service users, with more focus on personal coping strategies and more personal responsibility for the recovery process. Furthermore, Nyttingnes and Rugkåsa (2021) explored the different narratives that were recorded during the introduction of such medication-free mental health services. Their research compared the different discourses promoted by each stakeholder group: service providers, practitioners, service user led organisations, policy makers and the public. In this process there was a strong focus on the biomedical model and on an authoritarian stance that assumed the need for the sometime coercion of mental health medication; however, policy makers insisted on the cessation of all coercive mental health medication-based treatment (Oedegaard et al., 2020) drawing on the evidence base proffered by service user-led organisations (Nyttingnes & Rugkåsa, 2021). In support of this, Sugiura et al. (2020) have advocated the need to end coercion in mental health care and to develop a rights-based approach to mental health care in relation to the Convention on the Rights of Persons with Disabilities. They highlight the importance of involving service user experts in decision-making at all levels: in the micro relationship implemented in shared decision-making in decisions of individual treatment courses and in the development of policy at local and national levels; a focus we will return to.

The acknowledgement of the importance of lived experience and service user agency is often the very first step in implementing change in mental health services. Despite this, a process of change that moves from the biomedical model to a co-produced mental health service would require a system of mental health valuing lived experience at the centre of choices in treatment plans (Ramon et al., 2021) and the design of mental health services (DH, 2019). Innovative practices such as the Hearing Voices Network and Open Dialogue (Corstens and Schnackenberg, 2021) have the potential to occupy a subliminal space in which individuality can flourish. Romme and Escher (1993)’s ground-breaking research led to the development of the Hearing Voices Network (HVN), which unlike traditional therapies, concentrates on validating anomalous experiences (Baker, 2009). Treatment models often propound the eradication of voice-hearing as the focus of recovery as reinforced by Hui et al. (2018)’s study. Hui et al. (2018)’s conclusions about the importance of medication adherence, highlighted earlier, are limited because poor outcomes for patients who discontinued medication were defined solely in terms of a small difference in delusional symptomatology; factors which are positioned within a biomedical paradigm rather than a social model of mental health and recovery (Tew et al., 2012). Such a goal is rejected by advocates of the Hearing Voices Network (Romme & Escher, 1993; Romme et al., 2009) in which the objective of recovery (Baker, 2009) is to learn to live with voices, not necessarily eradicate them. Moreover, Hearing Voices groups (Lavender et al., 2017) utilise peer support to enable voice hearers to share coping mechanisms and to normalise these experiences within the construct developed by the individual. Additionally, Open Dialogue (OD), also a novel way of supporting people with mental ill-health (Seikula, 2011; Putman, 2021; Mosse et al., 2023), propounds a more limited use of mental health medication and emphasises the importance of the social network in managing the mental ill health of the person of concern (Steingard, 2021). It places talking in social networks as a precedent to taking medication and underlines the importance of communication and understanding of mental health causes. Common to both HVN (Coleman, 2021) and OD approaches (Nelson et al., 2021) is valuing expertise-by-experience. The application of such interventions raises questions about the way in which people with lived experience define their condition and the nature of their recovery, and its influence on their use of medication, life choices and self-identity.

This again returns me to raise questions about the validity of the recovery approach (Recovery in the Bin (RITB), 2016), notwithstanding the support many activists feel towards the original meaning of the concept itself. Indeed, it has been claimed (RITB, (nd) that the recovery approach (Deegan, 1996), which emerged in the 1990s, generated by the user movement, has lost its radical authenticity. RITB (nd) argues that recovery has been co-opted under a neoliberal definition which emphasises the societal need for people to work and be individually ‘recovered’ in a society based on individualism and meritocracy. RITB (2016) have developed an UnRecovery Star that acknowledges the structural elements of a neoliberal economy that reduce opportunities for people with mental health issues and are a barrier to their recovery. They note it is a social justice tool that can be utilised to call for action and change to promote equality of opportunity for people who are unrecovered, rejecting the notion of recovery which—they believe—has been co-opted by professionals, as alluded to in the introduction.

In my role as an academic and expert-by-experience, I have operated within a biomedical model that constrains recovery within the professional frameworks of practice and have found struggle and conflict in my own sense of self. King (2021) parallels my own conflict with identity as he reflects on his struggles between his roles as both a Minority Ethnic survivor of mental health services and a mental health social worker sometimes having to recommend compulsory admission to hospital. He highlights the realities of a discriminatory and oppressive mental health service in the UK—in which people of Colour, particularly men from Black African and Caribbean backgrounds, have been shown to be more likely than the majority White British population to be subjected to compulsory treatment (Gov.uk, 2022). King (2021) acknowledges the displacement he feels as he recognises this dichotomy between his authority as a mental health social worker and his identity as a person from a minority background.

Accordingly, service user activists and survivors have also challenged the identity of traditional mental health paradigms, defining their condition within the frame of the term neurodiversity. Neurodiversity is used to describe people’s individual and unique ways of viewing the world—none of which are designated as deficit based but used to acknowledge their diverse ways of being in the world. This concept emerged from the 1990s and has been adopted by people with autism (Kapp, 2020) and other neurological issues such as ADHD (Kuken, 2023). It is important to acknowledge this conceptual development in this article, but it is not possible to address it here in detail. However, recognition of such frameworks evidences the concept identified by Fricker (2007) of epistemic injustice. Within this paradigm, certain concepts or narratives are systematically neglected or discredited because of the social stereotypes associated with them. This can helpfully be applied to the experiences of mental health survivors, whose self-identification within the recovery model has been subsumed within the professional biomedical narrative. As such, my own self-concept has been negated by the discourse of biomedical approaches which privileges the knowledge of professionals above that of people with lived experience. Such an approach disavows the process of accepting and validating mental health knowledge within a survivor discourse, and thus personally disrupts my own self-concept. Thus, this article has formed an autoethnographic reflection of my own encounters with mental health and reinforces my own unique identity as a person who is in recovery, claiming back the place of lived experience in the mental health discourse (Fox, 2017).

## Conclusion

Through my reflections of lived experience using the framework of autoethnography I have considered my own mental distress and my treatment choices. I have addressed the application of both the medical and recovery models of mental health to my own life journey. Through this process I have connected the personal to the political, and the autobiographical to social systems (Ellis, 2004) to understand wider mental health policy. I have emphasised the importance of co-production and user involvement in wider mental health policy. This leads full circle to the questions I asked at the start: Have I become estranged from the roots of recovery by subscribing to the biomedical model of treatment? Has my academic identity challenged my expertise-by-experience?

Potentially the accusation by some survivor groups (RITB, nd) towards the co-option of the recovery approach into the neoliberal discourse, could parallel my own identity as I experience a displacement of my own lived experience from engaging in my professional social work research and teaching..As a social work educator, I engage with the implementation of social justice and human rights; elements at the centre of social work education and practice (Fook, 2016; Social Work England, (SWE) 2020). In challenge to the traditional paradigm of social work education, in which there is a strong divide between the personal and professional boundaries, I tell narratives of mental health recovery to both explore my own story and illuminate this often-hidden and stigmatised area of lived experience for social work students. As I break the silence that negates and denies the place of lived experience in social work practice, I explore this radical identity of lived experience with courage and conviction. This personal act marks a beginning as I acknowledge and challenge traditional models of mental health care and make space to own my own unique life story and life journey. Furthermore, this narrative enables social work students themselves to develop a sociological imagination (Wright Mills, 1959) by encountering "the awareness of the relationship between personal experience and the wider society” and connecting private troubles to public issues. This empowers them to reflect on and explore their own personal ethics and values alongside their sense of professionalism; a requirement of social work professional development (SWE, nd).

The lived experience narrative has been subsumed into a professional discourse (Fox, 2017) and recovery has been co-opted into a clinical framework (RITB, nd). Thus, the narrative explored in this article counters this discourse and accentuates the authenticity of lived experience, long captured in the disability movement’s demand for full involvement in society (Finkelstein, 1990), encapsulated in the challenge ‘Nothing about me without me!’ For many survivors, and for myself as a social work academic and person with lived experience, such a mantra extends to a demand for people accessing mental health services to be actively and fully involved in shared decision-making processes in their treatment choices, life options and valued based decisions. Thus, active participation is required in defining the narrative discourse (Fox, 2017) that underpins mental health philosophy, care and treatment.

## Footnotes

1 Social role valorisation and normalisation are concepts that emphasise the importance for service users of occupying valued and useful roles in society. This allows their identity to be expressed and valued in terms of their contribution to society, rather than devalued by their service user or disabled identity.
